# Bile Acid Dysregulation Is Intrinsically Related to Cachexia in Tumor-Bearing Mice

**DOI:** 10.3390/cancers13246389

**Published:** 2021-12-20

**Authors:** Morgane M. Thibaut, Justine Gillard, Adeline Dolly, Martin Roumain, Isabelle A. Leclercq, Nathalie M. Delzenne, Giulio G. Muccioli, Laure B. Bindels

**Affiliations:** 1 Metabolism and Nutrition Research Group, Louvain Drug Research Institute, Université Catholique de Louvain (UCLouvain), 1200 Brussels, Belgium; morgane.thibaut@uclouvain.be (M.M.T.); justine.gillard@uclouvain.be (J.G.); adeline.dolly@uclouvain.be (A.D.); nathalie.delzenne@uclouvain.be (N.M.D.); 2 Laboratory of Hepato-Gastroenterology, Institut de Recherche Expérimentale et Clinique, Université Catholique de Louvain (UCLouvain), 1200 Brussels, Belgium; isabelle.leclercq@uclouvain.be; 3 Bioanalysis and Pharmacology of Bioactive Lipids Research Group, Louvain Drug Research Institute, Université Catholique de Louvain (UCLouvain), 1200 Brussels, Belgium; martin.roumain@uclouvain.be (M.R.); giulio.muccioli@uclouvain.be (G.G.M.)

**Keywords:** ursodeoxycholic acid, bile flow, non-cachectic C26 mice, NC26, G protein-coupled bile acid receptor, GPBAR1, TGR5, TGR5 cell reporter assay, muscle atrophy, hepatic inflammation

## Abstract

**Simple Summary:**

Cancer cachexia is considered a multi-organ syndrome. An improved understanding of how circulating molecules can affect tissues and mediate their crosstalk in the pathogenesis of cancer cachexia is emerging. Considering the various actions of bile acids on host metabolism and immunity, they could represent innovative targets in cancer cachexia. In this study, we investigated how bile acids could contribute to this syndrome by assessing the bile flow, by comparing the impact on bile acid pathways of cachexia-inducing and non-cachexia-inducing cell sublines, and by investigating the effects of ursodeoxycholic acid, a choleretic compound, in cachectic mice. Altogether, our analyses strengthen the importance of bile acids and their receptors as key players in the metabolic disorders associated with cancer, thereby laying the foundation for new therapeutic opportunities.

**Abstract:**

Bile acids exert diverse actions on host metabolism and immunity through bile acid-activated receptors, including Takeda G protein-coupled receptor 5 (TGR5). We have recently evidenced an alteration in bile acids in cancer cachexia, an inflammatory and metabolic syndrome contributing to cancer death. This current study aims to further explore the links emerging between bile acids and cancer cachexia. First, we showed that bile flow is reduced in cachectic mice. Next, comparing mice inoculated with cachexia-inducing and with non-cachexia-inducing C26 colon carcinoma cells, we demonstrated that alterations in the bile acid pathways and profile are directly associated with cachexia. Finally, we performed an interventional study using ursodeoxycholic acid (UDCA), a compound commonly used in hepatobiliary disorders, to induce bile acid secretion and decrease inflammation. We found that UDCA does not improve hepatic inflammation and worsens muscle atrophy in cachectic mice. This exacerbation of the cachectic phenotype upon UDCA was accompanied by a decreased TGR5 activity, suggesting that TGR5 agonists, known to reduce inflammation in several pathological conditions, could potentially counteract cachectic features. This work brings to light major evidence sustaining the emerging links between bile acids and cancer cachexia and reinforces the interest in studying bile acid-activated receptors in this context.

## 1. Introduction

Cancer cachexia is a complex multi-organ syndrome characterized by unintentional weight loss, weakness, and muscle atrophy [1,2,3]. In addition, fat depletion, thermogenesis, and systemic inflammation were reported in clinical and/or preclinical models [4,5,6]. It affects up to 70% of cancer patients, depending on cancer type, and is responsible for at least 22% of cancer deaths [6,7,8]. Cancer cachexia does not only result in increased mortality rates but also in increased morbidity and reduced tolerance to anti-cancer treatments [8,9]. Cancer cachexia is a multi-organ syndrome driven, among other factors, by systemic inflammation and altered hormone production. Several proinflammatory mediators and tumor-derived catabolic factors are generated through tumor-immune crosstalk and have been shown to drive communication between the tissues such as tumor, muscle, adipose tissue, and liver [1,5,7]. Importantly, bile acids are well-known regulators of inflammation and energy homeostasis, two key features of cancer cachexia. Primary bile acids are synthesized in the liver from cholesterol. They are conjugated to taurine (mainly in mice) and glycine (mainly in humans). Conjugated primary bile acids are then concentrated in the gallbladder and secreted in the intestine, where they facilitate emulsification and absorption of dietary lipids and fat-soluble vitamins. In the intestine, a fraction of the bile acids will undergo bacterial metabolism, which includes deconjugation into free bile acids and transformation in secondary bile acids (e.g., through dehydroxylation) [10]. Most of these primary and secondary bile acids are reabsorbed in the distal ileum to the portal vein and reach the liver to complete the bile acid enterohepatic cycle [11]. There, secondary bile acids will be conjugated to glycine and taurine. A small amount of bile acids can also escape the liver uptake and reach the systemic circulation to act on peripheral organs and tissues. Once bile acids reach the tissues, they bind to several receptors and exert diverse actions on host metabolism and immunity [12,13]. Among these receptors, the transmembrane G protein-coupled bile acid receptor 1 (GPBAR1, also called TGR5) [14,15], is of particular interest in the context of cancer cachexia for two main reasons. First, TGR5 is expressed by several innate immune cells and controls inflammation in several pathological contexts [16,17,18,19,20]. Secondly, the activation of TGR5 in adipocytes and muscle cells leads to oxygen consumption and increased energy expenditure through the activation of the cAMP-dependent iodothyronine deiodinase 2 (Dio2) [21,22,23].

Cancer cachexia is largely characterized by systemic inflammation, including increased proinflammatory cytokines such as interleukin 1 (IL-1), interleukin 6 (IL-6), or tumor necrosis factor α (TNFα) [24,25]. Furthermore, it is generally accepted that inflammatory mediators affect the hepatobiliary transport system by a process termed “inflammation-induced cholestasis” [26,27,28]. Previous work from our team shows alterations in bile acid metabolism and hepatobiliary secretion in tumor-bearing mice with cachexia (C26 and LLC models) and in colorectal cancer patients with cachexia [29]. In this context, treatment of C26 mice with a neutralizing IL-6 antibody restored the expression of genes involved in the hepatobiliary transport, bile acid synthesis, and inflammation, demonstrating the causal role of IL-6 in the impairment of the hepatobiliary transport system. In addition, targeting bile acids using cholestyramine, a bile acid sequestrant, reduced hepatic inflammation without affecting the hepatobiliary transporters, highlighting the role played by bile acids in the hepatic inflammation [29]. Along with our findings, mild cholestasis has been documented in a cohort of cachectic and non-cachectic patients with various cancer types, characterized by an increase in serum levels of alkaline phosphatase and gamma glutamyl transpeptidase [30].

UDCA was historically used as a first-line medical treatment in chronic cholestatic diseases [31]. Today, UDCA is the only drug approved by the U.S. Food and Drug Administration (FDA) for the treatment of primary biliary cholangitis/cirrhosis (PBC), where it was shown to improve liver parameters and slow the disease progression [32,33]. However, the effectiveness of UDCA has often been controversial as many patients do not respond to the treatment [34]. The protective effects of UDCA appear to rely on its ability to stimulate hepatobiliary secretion, protect cholangiocytes against hydrophobic bile acids and protect hepatocytes against bile acid-induced apoptosis [35]. Since our previous study has shown alterations in the hepatobiliary transport system supporting inflammation-induced cholestasis in preclinical models and in cachectic cancer patients, and since UDCA displayed beneficial effects in hepatobiliary disorders, we hypothesized that treating C26 mice with UDCA could improve some hepatic inflammation, and thereby some cachectic features.

In the present work, we aimed to identify whether the alterations associated with bile acid metabolism previously observed in the C26 mouse model were related to cachexia or, more generally to the tumoral presence, using a non-cachexia-inducing C26 cell line. We also confirmed a decreased bile flow in the C26 cachectic mice, and we tested the hypothesis that UDCA, a choleretic compound, could represent an innovative strategy to alleviate cachectic features. Finally, we investigated how the modulation of the bile acid profile impacts the TGR5 activation potential.

## 2. Materials and Methods

### 2.1. Cell Culture

Cachexia-inducing colon carcinoma 26 (C26; a kind gift from Dr. Mario Colombo, Istituto Nazionale Tumori, Milan, Italy) and non-cachexia-inducing C26 colon carcinoma (NC) cells (TKG0518; obtained from the Cell Resource Center for Biomedical Research, Tohoku University) were maintained in DMEM high glucose medium supplemented with 10% fetal bovine serum (Capricorn Scientific, Ebsdorfergrund, Germany), 100 µg/mL streptomycin and 100 IU/mL penicillin (Thermo Fisher, Merelbeke, Belgium) at 37 °C with 5% CO_2_.

### 2.2. Mouse Experiments

Male CD2F1 mice (7 weeks old, Charles River Laboratories, Italy) were kept in specific pathogen-free conditions and housed in individually ventilated cages with a 12 h light/dark cycle and fed an irradiated chow diet (AO4-10, Safe, Augy, France). The model used to study cancer cachexia is the well-established C26 model, characterized by body weight and fat mass loss as well as muscle atrophy [36,37,38]. After one week of acclimatization, mice were randomly assigned to experimental groups based on their body weight and were subcutaneously injected in the upper flank with a saline solution, C26, or NC cells (1 × 10^6^ cells in 0.1 mL saline). For the UDCA experiment, mice received 300 mg/kg of a suspension of ursodeoxycholic acid (Sigma-Aldrich, St. Louis, MO, USA) in methylcellulose and glycerol 10% or vehicle by oral gavage [39], from day 1 after cell injection until the end of the experiment (eight mice per group). Food intake and body weight were recorded. Ten days after cancer cell injection, mice were fasted for 6 h, and tissue samples were harvested following anesthesia (isoflurane gas, Abbott, Wavre, Belgium). Tissues were weighed and frozen in liquid nitrogen. All samples were stored at −80 °C until further analyses.

For the bile flow measurement, CT and C26 mice were fasted for 6 h and were anesthetized under isoflurane gas. The gallbladder was cannulated after common bile duct ligation for the collection of bile. After a 10 min equilibration period, bile was collected in pre-weighed tubes for 30 min, and bile flow was determined gravimetrically and normalized to liver weight. The liver was weighed and frozen in liquid nitrogen. We obtained results for 4 CT and 8 C26 mice out of 10 mice per group due to mortality during the bile collection.

### 2.3. TGR5 Activation in a Cell Reporter Assay

The principle of the assay is the following. Activation of TGR5 promotes the adenylyl cyclase cAMP signaling pathway, leading to the activation of cAMP response element-binding protein that will promote the transcription of the firefly luciferase. Of note, this assay reflects the activation potential of TGR5 by individual bile acids or blood samples. The signaling downstream of TGR5 has been shown to be, to some extent, cell-specific [40]; this assay does not allow extrapolation about the extent of activation of secondary messengers, which is a cell-specific feature. HEK293T cells (ATCC CRL-3216) were cultured in DMEM medium containing 10% FBS and 1% penicillin/streptomycin at 37 °C and 5% of CO_2_. At 80% of confluence in a 96-well plate, cells were transfected with 20 ng of pCMV-SPORT6 human TGR5/GPBAR1 (Harvard Medical School MGC:40597), 40 ng of pGL4.29 (CRE-luciferase, Promega, Madison, WI, USA), and 5 ng of pGL4.73 (SV40-Renilla, Promega, Madison, WI, USA) using Lipofectamine 2000. Twenty-four hours after transfection, cells were incubated with FBS-free medium (CTL) or FBS-free medium containing 10 µM of tauro-lithocholic acid (TLCA), tauro-cholic acid (TCA), tauro-chenodeoxycholic acid (TCDCA), tauro-α-muricholic acid (Tα-MCA), tauro-β-muricholic acid (Tβ-MCA), tauro-deoxycholic acid (TDCA) and tauro-ursodeoxycholic acid (TUDCA) or 10% portal plasma for 3 h. Then, cells were lysed and assayed according to the Dual-Luciferase Reporter Assay System (Promega E1910). Firefly and renilla luminescences were quantified using a GloMax 20/20 Luminometer (Promega, Leiden, The Netherlands). Firefly luciferase signal was normalized to renilla luciferase signal as an internal control of the transfection efficiency. The signal is TGR5-dependent as no signal was detected in cells transfected only with pGL4.29 and pGL4.73, without the pCMV-SPORT6 human TGR5/GPBAR1 (Figure S1).

### 2.4. Bile Acid Quantification

Bile acid quantification was performed by HPLC-MS as previously described [41]. Briefly, liver samples were homogenized in ice-cold distilled water, and proteins were precipitated using acetone in the presence of deuterated internal standards. The samples were next centrifuged, the supernatant recovered and evaporated to dryness. The resulting residue was resuspended in methanol and analyzed by HPLC-MS using an LTQ-Orbitrap XL coupled to an Accela HPLC system (Thermo Fisher, Merelbeke, Belgium). Analyte separation was performed on an Ascentis Express C-18 column (2.7 µm, 4.6 × 100 mm) (Sigma-Aldrich, St. Louis, MI., USA). The separation was achieved using a gradient of H_2_O-ACN-formic acid 75:25:0.1 (*v*/*v*/*v*) and ACN-formic acid 100:0.1 (*v*/*v*). The MS analysis was performed in the negative mode with an ESI ionization source. Calibration curves were prepared using the same conditions. Data are expressed as pmol normalized by the amount of tissue. Values below the LOQ (for data sets with less than 25% of such missing values) were imputed using the function *impute.QRILC* in the R package *imputeLCMD* [42].

### 2.5. Tissue mRNA Analysis

Total RNA was isolated from the tissue by TriPure reagent (Roche, Basel, Switzerland). cDNA was prepared by reverse transcription of 1 μg total RNA using the Goscript RT Mix OligoDT kit (Promega, Leiden, The Netherlands). Real-time polymerase chain reactions (PCR) were performed with a StepOnePlus/QuantStudio Real-Time PCR System and software (Applied Biosystems, Den Ijssel, The Netherlands) or a CFX96 TouchTM instrument and software (Bio-Rad Laboratories, Hercules, CA, USA; version 3.1) using SYBR Green (Applied Biosystems, Promega, Seraing, Belgium) for detection. All samples were run in duplicate in a single 96-well reaction plate, and data were analyzed according to the 2^−ΔΔCT^ method. The purity of the amplified product was verified by analyzing the melt curve performed at the end of amplification. The ribosomal protein L6 (*Rpl6*) gene was used as a housekeeping gene, with the exception of the brown adipose tissue where the ornithine decarboxylase antizyme 1 (*Oaz1*) was selected as a housekeeping gene. The primer sequences for the targeted mouse genes are detailed in Table S1.

### 2.6. Statistical Analyses

Data were analyzed using a Student *t*-test when comparing two groups, one-way ANOVA followed by Bonferroni’s pairwise comparison post-hoc tests with the C26 group as reference for the NC study, or two-way ANOVA with Bonferroni’s pairwise comparison post-hoc tests comparing the mice treated with the vehicle or UDCA in the CT groups and C26 groups for the UDCA study. All data were checked for normality using the Shapiro–Wilk normality test. Data determined to be non-normal even after log-transformation were analyzed using a Mann–Whitney *U* test or Kruskal–Wallis test with Bonferroni’s post-tests. Outliers were identified using the Grubb’s test and removed. Correlation analyses were performed using Spearman correlations. Statistical analyses were carried out using GraphPad Prism 8.0 (GraphPad Software, San Diego, CA, USA). *p* < 0.05 was considered statistically significant.

## 3. Results

### 3.1. Decreased Bile Flow and Alterations in the Hepatobiliary Transport System in C26 Cachectic Mice

**Figure 1:**
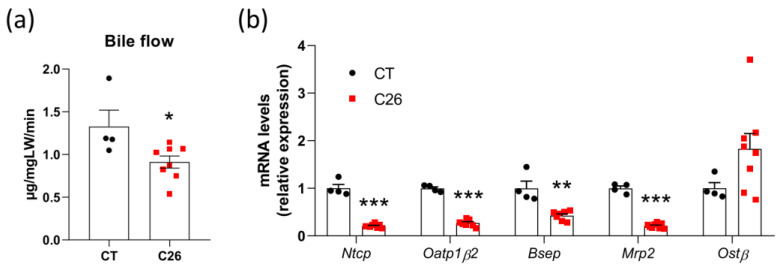
Decreased bile flow and alterations in the hepatobiliary transport system in C26 cachectic mice. (**a**) Bile flow in sham-injected mice (CT; *n* = 4) and colon carcinoma 26-transplanted mice (C26; *n* = 8). (**b**) Hepatic mRNA expression levels of genes involved in the hepatobiliary transport system in CT and C26 mice. *Ntcp*, Na(+)/taurocholate transport protein; *Oatp1β2*, organic anion transporter family member 1B2; *Bsep*, bile salt export pump; *Mrp2*, multidrug resistance-associated protein 2; *Ostβ*, organic solute transporter subunit beta. *n* = 4–8 mice per group; data are presented as mean ± SEM, * *p* < 0.05, ** *p* < 0.01, *** *p* < 0.001.

We have previously shown alterations in bile acid metabolism and signs of an impaired hepatobiliary transport system in C26 cachectic mice [29]. We, therefore, hypothesized that decreased gene expression of the hepatobiliary transporters could reduce the bile secretion and thereby hamper the bile flow. We thus measured the bile flow in C26 cachectic mice (C26) as compared to sham-injected mice (CT). We found a significant decrease in the bile flow of C26 mice in line with a decreased hepatic expression of genes involved in bile acid uptake (*Ntcp* and *Oatp1β2*) and bile acid secretion (*Bsep* and *Mrp2*), and an increased expression of *Ostβ*, a gene involved in the alternative bile acid efflux (Figure 1a,b). These data confirm an impairment in the bile flow that could contribute to the modifications of the bile acid profile and could contribute to cancer cachexia.

### 3.2. Many Alterations in the Liver, Brown Adipose Tissue and Muscle Are Intrinsically Related to Cachexia in the C26 Model

**Figure 2:**
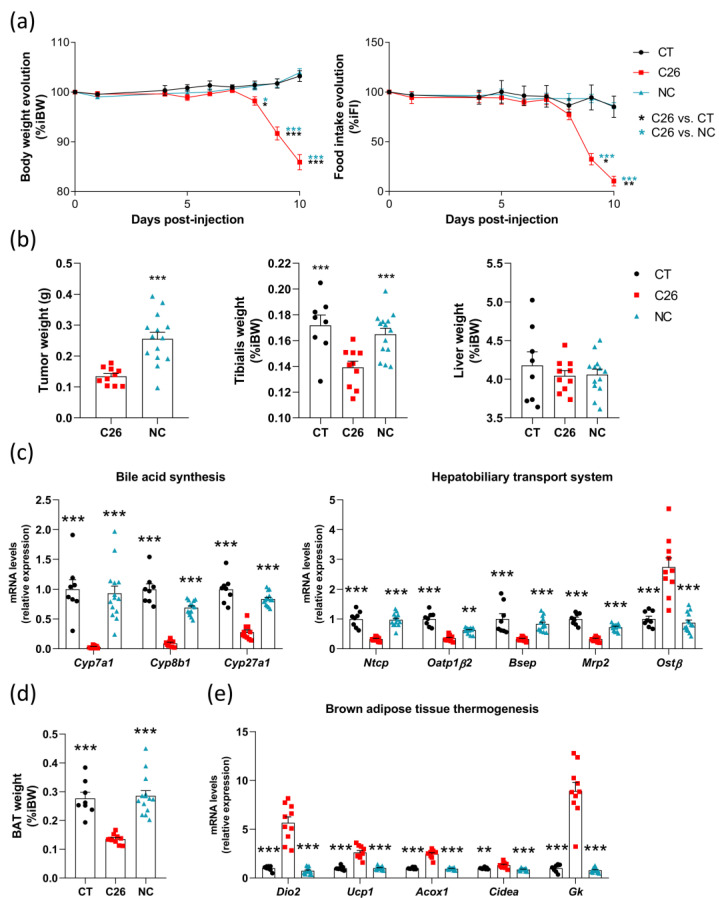
Many alterations in the liver, muscle, and brown adipose tissue are intrinsically related to cachexia and not to the tumor in C26 mice. (**a**) Body weight and food intake evolution in sham-injected mice (CT; *n* = 8), mice injected with cachexia-inducing C26 colon carcinoma cells (C26 mice; *n* = 10), and mice injected with non-cachexia-inducing C26 colon carcinoma cells (NC mice; *n* = 14), expressed in % of initial body weight or food intake. (**b**) Tumor, tibialis, and liver weights of CT, C26, and NC mice. (**c**) Hepatic mRNA expression levels of genes involved in the bile acid synthesis and the hepatobiliary transport system in CT, C26, and NC mice. (**d**) The brown adipose tissue weight of CT, C26, and NC mice. (**e**) mRNA expression levels of genes involved in thermogenesis in the brown adipose tissue of CT, C26, and NC mice. *Cyp7a1*, cytochrome P450 family 7 sub-family A member 1; *Cyp8b1*, cytochrome P450 family 8 sub-family B member 1; *Cyp27a1*, cytochrome P450 family 27 sub-family A member 1; *Ntcp*, Na(+)/taurocholate transport protein; *Oatp1β2*, organic anion transporter family member 1B2; *Bsep*, bile salt export pump; *Mrp2*, multidrug resistance-associated protein 2; *Ostβ*, organic solute transporter subunit beta; *Dio2*, iodothyronine deiodinase 2; *Ucp1*, uncoupling protein 1; *Acox1*, acyl-coA oxidase 1; *Cidea*, cell death-inducing DFFA-like effector A; *Gk*, glycerol kinase. Data are presented as mean ± SEM, * *p* < 0.05, ** *p* < 0.01, *** *p* < 0.001 vs. C26.

Next, we wanted to determine whether the alterations we observed in C26 mice were related to cachexia in particular or, more generally, to the tumoral presence. For this purpose, we compared mice inoculated with cachexia-inducing C26 colon carcinoma cells (C26 mice; *n* = 10), non-cachexia-inducing C26 colon carcinoma cells (NC mice; *n* = 14), and sham-injected mice (CT mice; *n* = 8). All mice were necropsied on the same day. We did not observe any weight loss or reduction in food intake in NC and CT mice, as opposed to C26 mice (Figure 2a). The tumor weight on the day of necropsy was two-fold higher in NC mice, and no reduction in tibialis muscle weight was observed in NC mice, whereas it was reduced by 19% in C26 mice (Figure 2b). Liver weight was not affected (Figure 2b). In the liver, we confirmed for C26 mice the alterations in the expression levels of genes involved in bile acid synthesis *(Cyp7a1*, *Cyp8b1*, and *Cyp27a1)* and of genes controlling the hepatobiliary transport system *(Ntcp*, *Oatp1β2*, *Bsep*, *Mrp2*, and *Ostβ*) (Figure 2c). Importantly, the NC mice displayed similar expression levels of these genes as in CT mice. Regarding the brown adipose tissue, C26 mice showed a reduced tissue weight and an induction of the expression of genes involved in thermogenesis *(Dio2*, *Ucp1*, *Acox1*, *Cidea*, and *Gk*) (Figure 2d,e). No reduction in the brown adipose tissue weight and no change in key markers of thermogenesis were observed in NC mice (Figure 2d,e). Together, these results show that these alterations in the liver, muscle, and brown adipose tissue are intrinsically related to cachexia and not only due to the presence of the tumor in the C26 model.

### 3.3. Alterations in Bile Acid Profile Are Intrinsically Related to Cachexia without Any Modification of TGR5 Activation Capacity in C26 Cachectic Mice

**Figure 3:**
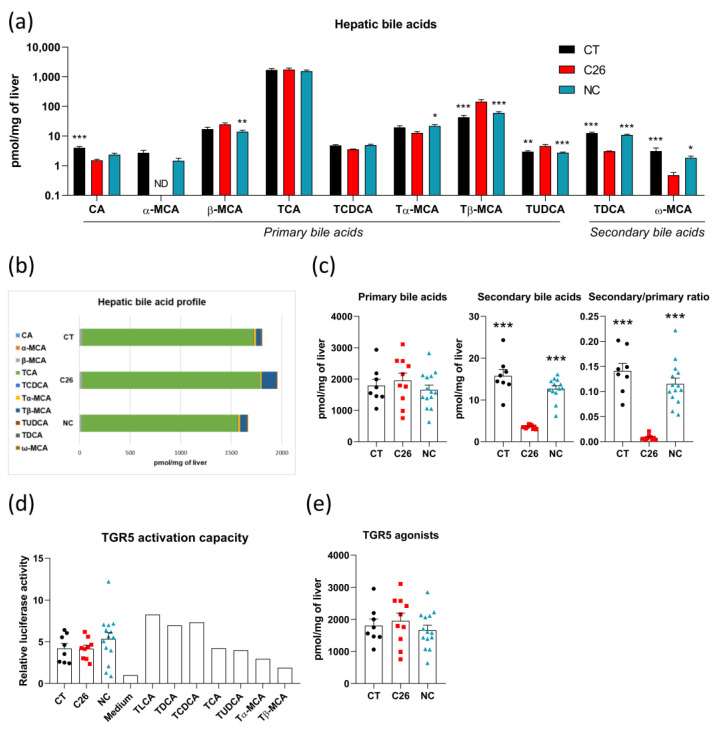
Alterations in bile acid profile are intrinsically related to cachexia without any modification of TGR5 activation capacity in C26 mice. (**a**) Hepatic bile acids in sham-injected mice (CT; *n* = 8), mice injected with cachexia-inducing C26 colon carcinoma cells (C26 mice; *n* = 10), and mice injected with non-cachexia-inducing C26 colon carcinoma cells (NC mice; *n* = 14). (**b**) Hepatic bile acid profile in CT, C26, and NC mice. (**c**) Primary and secondary bile acid levels and Secondary/primary ratio in the liver of CT, C26, and NC mice. (**d**) TGR5 activation capacity using cell reporter assay incubated with portal plasma of CT, C26, and NC mice or 10 µM of bile acids. (**e**) TGR5 agonists (including CA, TCA, TCDCA, TUDCA, and TDCA) levels in the liver of CT, C26, and NC mice. ND, Not detected; CA, cholic acid; α-MCA, α-muricholic acid; β-MCA, β-muricholic acid; TCA, tauro-cholic acid; TCDCA, tauro-chenodeoxycholic acid; Tα-MCA, tauro-α-muricholic acid; Tβ-MCA, tauro-β-muricholic acid; TUDCA, tauro-ursodeoxycholic acid; TDCA, tauro-deoxycholic acid; ω-MCA, ω-muricholic acid. CDCA, DCA, lithocholic acid (LCA), and TLCA were undetected. Data are presented as mean ± SEM, * *p* < 0.05, ** *p* < 0.01, *** *p* < 0.001 vs. C26.

Hepatic bile acid profiling revealed that the levels of several bile acids were strongly altered in C26 mice as compared to CT mice, whereas they were not changed in NC mice, including primary bile acids, namely α-muricholic acid (α-MCA), tauro-β-muricholic acid (Tβ-MCA), tauro-ursodeoxycholic acid (TUDCA) and secondary bile acids namely tauro-deoxycholic acid (TDCA) and ω-muricholic acid (ω-MCA) (Figure 3a). In addition, we found a significant decrease in cholic acid (CA) in C26, as compared to CT mice, as well as a decrease in β-muricholic acid (β-MCA) and an increase in tauro-α-muricholic acid (Tα-MCA) in NC mice as compared to C26 mice (Figure 3a,b). Interestingly, even though total primary bile acid levels were not altered between groups, C26 cachectic mice were characterized by a four-fold decrease in total secondary bile acids, resulting in an alteration of the secondary/primary ratio (Figure 3c). These changes were not observed in NC mice.

As mentioned earlier, bile acids can act on the TGR5 receptor and activate signaling pathways in immune cells, liver, brown adipose tissue, and muscle, thereby controlling metabolic and inflammatory processes [15]. LCA and DCA, as well as their tauroconjugates, are recognized as the strongest TGR5 agonists [12,37]. Of note, LCA derivatives were not detected in these mice. As TDCA was reduced in C26 mice, we decided to investigate whether the TGR5 activation capacity was also reduced in these mice. To do so, we quantified the TGR5 activation potential of the portal plasma of CT, C26, and NC mice using a cell reporter assay. To complement our analysis and better determine to what extent each bile acid contributes to the TGR5 activation, we also incubated reporter cells with several bile acids individually. Unexpectedly, we found no significant difference in TGR5 activation capacity between the CT, C26, and NC groups (Figure 3d). Moreover, in accordance with previous reports [12,13], we identified that the main TGR5 agonists were tauro-lithocholic acid (TLCA) and TDCA, the two secondary bile acids mentioned above, but also tauro-chenodeoxycholic acid (TCDCA), TCA, and TUDCA (Figure 3d), which are primary bile acids. This observation likely explains why the TGR5 activation capacity is not affected in C26 mice despite the reduction in secondary bile acids. Indeed, in line with the TGR5 activation capacity, we did not observe change in hepatic TGR5 agonist levels between CT, C26, and NC mice (Figure 3e). Along these lines, a significant positive correlation appeared between the portal TGR5 activation capacity and the hepatic levels of TGR5 agonists in these mice (Spearman rho = 0.45, *p* = 0.0096) (Figure S2). Altogether, these results highlight that bile acid profile alterations are associated with cachexia itself, cannot only be ascribed to the presence of the tumor and do not translate into a modification of the TGR5 activation capacity.

### 3.4. UDCA Treatment Changes the Bile Acid Profile and Decreases TGR5 Activation Capacity in C26 Cachectic Mice

**Figure 4:**
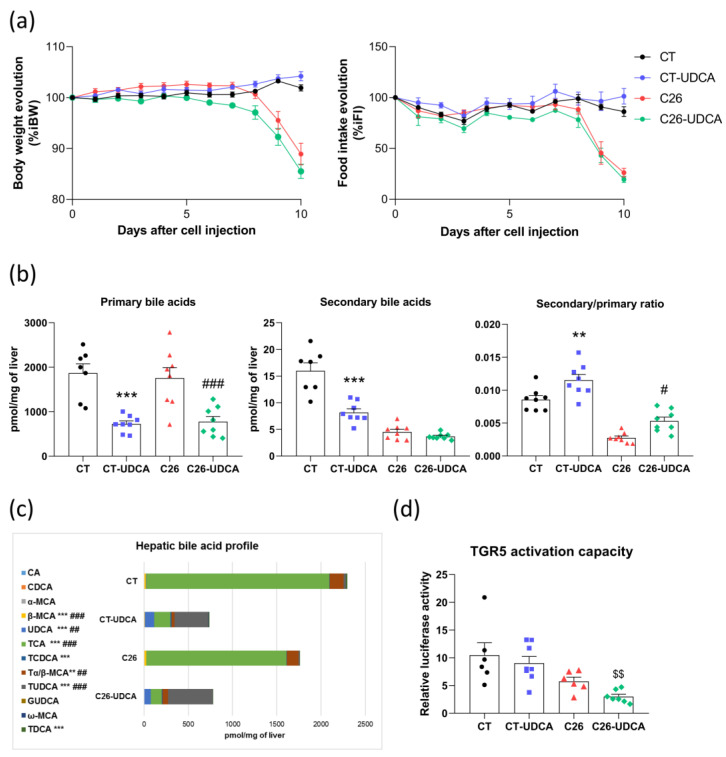
UDCA treatment changes the bile acid profile and decreases TGR5 activation capacity in C26 cachectic mice. (**a**) Body weight and food intake evolution in sham-injected mice (CT), sham-injected mice treated with UDCA (CT-UDCA), mice injected with cachexia-inducing C26 colon carcinoma cells (C26), and mice injected with cachexia-inducing C26 colon carcinoma cells and treated with UDCA (C26-UDCA). (**b**) Primary and secondary bile acid levels and Secondary/primary ratio in the liver of CT, CT-UDCA, C26, and C26-UDCA mice. (**c**) Hepatic bile acid profile in CT, CT-UDCA, and C26 and C26-UDCA mice. (**d**) TGR5 activation capacity using cell reporter assay incubated with portal plasma of CT, CT-UDCA, C26, and C26-UDCA mice. *n* = 5–8 mice per group; data are presented as mean ± SEM. ** *p* < 0.01, *** *p* < 0.001 CT vs. CT-UDCA and # *p* < 0.005, ## *p* < 0.01, ### *p* < 0.001 C26 vs. C26-UDCA. Student *t*-test C26 vs. C26-UDCA, $$ *p* < 0.01.

UDCA is a choleretic compound exerting beneficial effects on liver parameters and slowing the progression of chronic cholestatic diseases. Since our previous study has shown alterations in the hepatobiliary transport system and that bile flow is reduced in the C26 cachectic mice, we hypothesized that treating C26 mice with UDCA could improve some hepatic cachectic features, and thereby also some extra-hepatic cachectic features. To evaluate the impact of this compound on cancer cachexia, we treated CT and C26 mice with UDCA (daily gavage of 300 mg/kg). UDCA treatment did not affect body weight and food intake evolution within the CT and C26 groups (Figure 4a). UDCA treatment also had no effect on tumor and brown adipose tissue weights but significantly decreased the liver weight in C26 mice (Figure S3a). Moreover, UDCA treatment strongly reduced total hepatic bile acid levels in CT and C26 mice, suggesting an activation of the bile acid secretion, and deeply modified the hepatic bile acid profile (Figure 4b,c and Figure S3b). The treatment reduced total primary bile acid levels in both CT and C26 mice, whereas it had no effect on total secondary bile acid levels in C26 mice, which were already decreased, thereby leading to an increase in the secondary/primary ratio in CT and C26 mice (Figure 4b). Regarding the bile acid profile, it shifted from a profile mainly composed of TCA and Tα/β-MCA to a profile dominated by UDCA and derivatives in CT-UDCA and C26-UDCA groups, without affecting levels of TCDCA and TDCA in C26-UDCA mice (Figure 4c). Interestingly, the portal TGR5 activation capacity was decreased in C26-UDCA as compared to C26 mice (Student t-test *p* = 0.0068, Figure 4d). Altogether, these results revealed that UDCA treatment profoundly changed the bile acid profile resulting in a decreased TGR5 activation potential in C26 mice.

### 3.5. UDCA Treatment Does Not Improve Hepatic Inflammation and Exacerbates Muscle Atrophy in C26 Cachectic Mice

**Figure 5:**
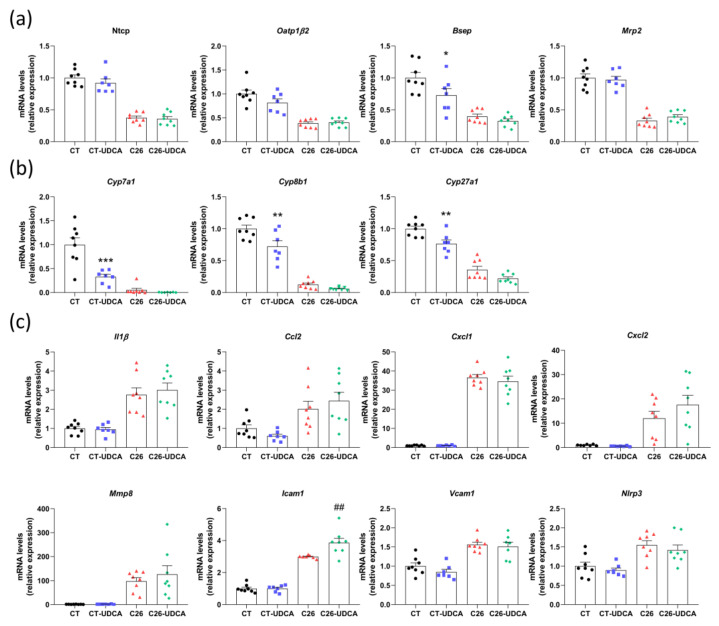
UDCA treatment does not improve hepatic inflammation in C26 cachectic mice. Hepatic mRNA expression levels of genes involved in the hepatobiliary transport system (**a**), bile acid synthesis (**b**) and inflammation (**c**) in sham-injected mice (CT), sham-injected mice treated with UDCA (CT-UDCA), mice injected with cachexia-inducing C26 colon carcinoma cells (C26), and mice injected with cachexia-inducing C26 colon carcinoma cells and treated with UDCA (C26-UDCA). *Ntcp*, Na(+)/taurocholate transport protein; *Oatp1β2*, organic anion transporter family member 1B2; *Bsep*, bile salt export pump; *Mrp2*, multidrug resistance-associated protein 2; *Cyp7a1*, cytochrome P450 family 7 sub-family A member 1; *Cyp8b1*, cytochrome P450 family 8 sub-family B member 1; *Cyp27a1*, cytochrome P450 family 27 sub-family A member 1; *Il1β*, interleukin-1β; *Ccl2*, C-C motif chemokine ligand 2; *Cxcl1*, C-X-C motif chemokine ligand 1; *Cxcl2*, C-X-C motif chemokine ligand 2; *Mmp8*, matrix metallopeptidase 8; *Icam1*, intercellular adhesion molecule 1; *Vcam1*, vascular cell adhesion molecule 1; *Nlrp3*, NLR family pyrin domain containing 3. *n* = 7–8 mice per group; data are presented as mean ± SEM. * *p* < 0.05, ** *p* < 0.01, *** *p* < 0.001 CT vs. CT-UDCA and ## *p* < 0.01 C26 vs. C26-UDCA.

We next evaluated the impact of UDCA treatment on hepatic gene expression. The expression of genes involved in the hepatobiliary transport system *(Ntcp*, *Oatp1β2*, *Bsep*, and *Mrp2)* or bile acid synthesis *(Cyp7a1*, *Cyp8b1*, and *Cyp27a1)* was not changed in C26-UDCA mice (Figure 5a,b). One of the consequences of cholestasis is the induction of proinflammatory cytokines, as well as recruitment of neutrophils in the liver of C26 cachectic mice [29,43]. Therefore, we analyzed the expression of genes involved in inflammation *(Il1β* and *Nlrp3)* and genes involved in neutrophil recruitment and adhesion *(Ccl2*, *Cxcl1*, *Cxcl2*, *Mmp8*, *Icam1*, and *Vcam1)*. Hepatic inflammation was not improved and rather tended to be aggravated in C26-UDCA mice, with a significant increase in *Icam1* expression (Figure 5c).

**Figure 6:**
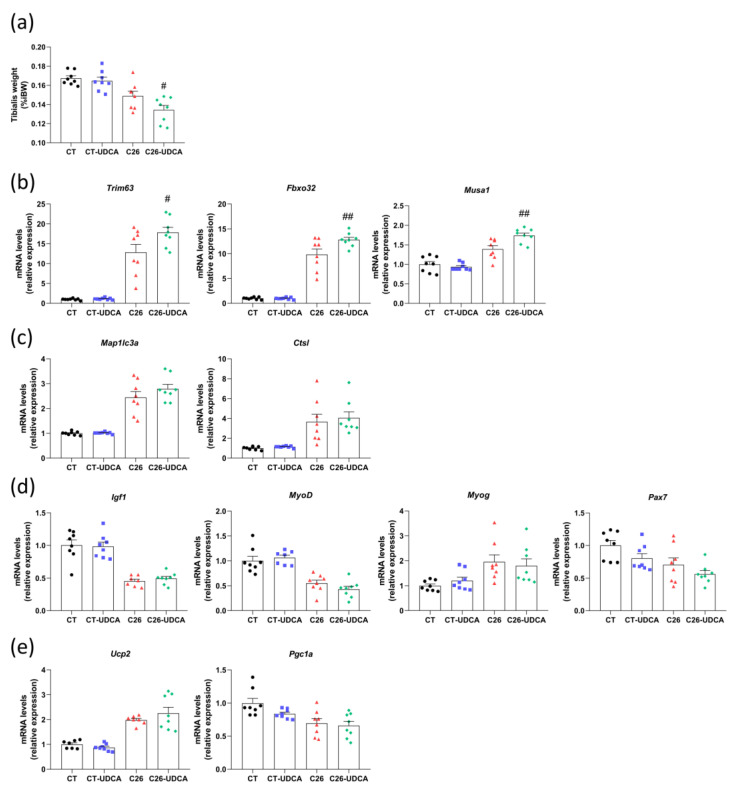
UDCA treatment worsens muscle atrophy in C26 cachectic mice. (**a**) Tibialis weight in sham-injected mice (CT), sham-injected mice treated with UDCA (CT-UDCA), mice injected with cachexia-inducing C26 colon carcinoma cells (C26), and mice injected with cachexia-inducing C26 colon carcinoma cells and treated with UDCA (C26-UDCA). Tibialis mRNA expression levels of genes involved in the ubiquitin-proteasome pathway (**b**), autophagy-lysosome pathway (**c**), differentiation (**d**), and mitochondrial function (**e**). *Trim63*, tripartite motif-containing 63 (also known as Murf1); *Fbxo32*, F-box protein 32 (also known as Atrogin1); *Musa1*, muscle ubiquitin ligase of the SCF complex in atrophy 1; *Map1lc3a*, microtubule-associated protein 1 light chain 3 alpha; *Ctsl*, cathepsin L; *Igf1*, insulin-like growth factor 1; *Myog*, myogenin; *MyoD*, myogenic differentiation 1; *Pax7*, paired box 7; *Ucp2*, uncoupling protein 2; *Ppargc1a*, PPARG coactivator 1 alpha. *n* = 7–8 mice per group; data are presented as mean ± SEM. # *p* < 0.05 ## *p* < 0.01 C26 vs. C26-UDCA.

Furthermore, we observed a decrease in the tibialis weight in C26-UDCA mice compared to untreated C26 mice (Figure 6a). This muscle mass loss was consistent with an increase in the expression of *Trim63*, *Fbxo32*, and *Musa1* (involved in the ubiquitin-proteasome pathway) (Figure 6b), while *Map1lc3a* and *Ctsl* (involved in the autophagy-lysosome pathway) were not significantly affected (Figure 6c). As TGR5 activation capacity was shown to foster differentiation and promote the expression of *Igf1* in TGR5-overexpressing muscle cells [44], we measured the expression of *Igf1* (a major inducer of myogenic cell proliferation), *MyoD*, and *Myog* (markers of muscle differentiation), and *Pax7* (a master regulator of satellite cell function) in the tibialis of these mice. None of these markers were affected by the UDCA treatment (Figure 6d). *Ucp2* and *Ppargc1a* were also measured as markers of mitochondrial biogenesis and bioenergetic expense mitochondrial biogenesis, but none of these markers were affected by UDCA treatment (Figure 6e). Altogether, this last set of analyses demonstrates that UDCA treatment does not improve hepatic inflammation and even worsens muscle atrophy in C26 cachectic mice.

## 4. Discussion

Cancer cachexia is currently considered as a multi-organ syndrome, and understanding of how new circulating molecules can affect tissues in the pathogenesis of cancer cachexia is emerging [1,5,7,8]. Considering the various actions of bile acids on host metabolism and immunity [12] and the modulation of their levels in cancer cachexia [29], bile acids appear as an interesting lead in this pathophysiological context. Therefore, in this study, we explored the links between bile acids and cancer cachexia by assessing the bile flow, by comparing the impact of cachexia-inducing and non-cachexia-inducing cell sublines on bile acid pathways, and by investigating the effects of UDCA, a choleretic compound, in cachectic mice. Limitations of our work include the use of one mouse model, which although being widely used and accepted in the cachexia field, implies an ectopic tumor, and the lack of measurement of the bile flow in NC and UDCA-treated mice.

Our previous work reports deep alterations in the bile acid pathways in cachectic mice and patients. In this context, we showed, using pair-feeding, that reduced food intake does not drive bile acid alterations in C26 mice [29]. Here, we wanted to decipher whether bile acid-associated alterations observed in C26 mice are related to cachexia or, more generally, to the tumoral presence. By comparing mice injected with the common cachexia-inducing C26 cells (C26) to mice inoculated with the non-cachexia-inducing C26 cells (NC), we confirm that many alterations in the liver, muscle, and brown adipose tissue are intrinsically related to cachexia and cannot be attributed only to the presence of the tumor in the C26 model. One potential explanation for these differences in cachectic phenotype could arise from the higher circulating levels of IL-6 in C26 mice as compared to NC mice, as reported in Reddel et al. [45]. Furthermore, the hepatic bile acid profile highlights a decrease in total secondary bile acids (especially for TDCA), arising from bacterial transformation, occurring only in C26 cachectic mice. Consistent with our previous work showing that the gut microbiota appears as a novel actor in cancer cachexia [46,47], and based on our knowledge of the bile acids-microbiota crosstalk [10,11,48], we speculate that the disruption in the hepatobiliary secretion, supported here by the functional measurement of the bile flow, may also contribute to the gut bacterial dysbiosis found in cancer cachexia. Vice versa, gut bacterial dysbiosis may contribute to the altered bile acid profile. Together, these data reinforce the interest in studying the crosstalk between bile acids and the microbiota in this context.

Cholestasis was described in a few specific cases of paraneoplastic conditions. Impairment of bile secretion appeared in Stauffer’s syndrome, a rare complication occurring in patients with renal carcinoma, and in a limited number of case reports in paraneoplastic conditions in Hodgkin’s lymphoma and prostate carcinoma [49,50,51]. In the present work, we confirm an impairment of bile flow in C26 cachectic mice. Bile flow is an osmotic process, where the water flow is partially driven by a solute concentration gradient that depends on primary active transporter pumps [52,53,54]. An impairment of the function of these transporters has a direct consequence on the bile secretion and therefore could contribute to cancer cachexia in several ways. (i) The main function of the bile salts is to emulsify dietary lipids and fat-soluble vitamins for absorption. Such impaired bile acid secretion in cachectic cancer patients could have serious consequences on lipid and fat-soluble vitamin digestion and might thereby worsen the cachectic phenotype. (ii) Bile is the major route for excretion of xenobiotic and potentially toxic lipophilic compounds, including several antineoplastic agents [55]. It is already recommended to adapt the doses of antineoplastic drugs in cancer patients with impaired liver function [56], which could further reduce the tolerance to the treatment of cachectic patients.

UDCA is well known for its choleretic potential. In the present study, UDCA treatment induces a bile acid profile dominated by UDCA and derivatives. It also decreases the total hepatic bile acid levels in CT-UDCA and C26-UDCA mice, strongly suggesting the activation of the bile acid secretion. Underlying mechanisms remain incompletely elucidated, but TUDCA was shown to stimulate the translocation of the key bile acid transporters BSEP and MPR2 into the canalicular membrane through activation of mitogen-activated protein kinases (Erk1/2 and p38^MAPK^) and integrin-dependent mechanisms in rat hepatocytes [57,58]. Other reports showed that the choleretic effect of TUDCA is mediated by increased Ca^++^ intracellular concentration and activation of protein kinase C/A-dependent signaling in cholestatic rat liver [59,60,61]. These findings, as well as our own observations showing no change in gene expression of hepatobiliary transporters in C26-UDCA mice, suggest that the choleretic effect of UDCA relies on its capacity to modulate transporters at the post-transcriptional level rather than at the transcriptional level [62,63]. Moreover, we showed previously that IL-6 is the main driver of the decreased gene expression of the hepatobiliary transporters in the C26 model, suggesting that the UDCA treatment is not effective enough to antagonize the effect of IL-6 [29].

One intriguing finding is the reduction in TGR5 activation capacity upon UDCA treatment, specifically in C26 mice. To identify which bile acids could be explaining this finding, we compared the impact of UDCA on the hepatic bile acid profile in CT and C26 mice, focusing on TDCA, TCDCA, TUDCA, and TCA, which are the main bile acids at play here to determine TGR5 activation capacity. We found that TCDCA was increased upon UDCA treatment in CT mice but not in C26 mice. This lack of change in TCDCA levels, a strong TGR5 agonist (Figure 3d), in C26 mice, may explain why UDCA treatment led to a lower TGR5 activation capacity in C26 mice compared to CT mice.

UDCA is known for its anti-inflammatory and cytoprotective activities and has been proposed as an interesting drug in the prevention and treatment of cancer [64]. However, its effects are controversial in cholestatic diseases. In our study, hepatic inflammation was not improved and rather tended to be aggravated in C26-UDCA mice, with a significant increase in *Icam1* expression. In line with our results, many studies reported poor beneficial effects of UDCA in several mouse models of cholestasis. In *Mdr2*^-/-^ mice, a model of primary sclerosing cholangitis, UDCA feeding showed an antifibrotic effect while worsening bile infarcts [65,66]. In bile duct-ligated mice, UDCA treatment increased hepatocyte necrosis and bile infarcts [65,67]. One explanation could be that when there is a serious or complete biliary obstruction, the beneficial effects of UDCA are lost due to excessive biliary pressure. Clinically, UDCA has higher beneficial effects in primary sclerosing cholangitis patients when it is combined with endoscopic treatment, suggesting that maintenance of the bile flow is essential for an efficient UDCA therapy [68,69]. However, in the C26 model, the bile flow is not obstructed, implying that this is probably not the cause of hepatic inflammation.

Another possible explanation relies on the decreased TGR5 activation capacity, whose anti-inflammatory potential is well known [16,17]. In Kupffer cells, the activation of TGR5 downregulates the expression of proinflammatory chemokines through several signaling pathways, including decreased NfκB transcriptional activity and inhibition of the NLRP3 inflammasome [70,71,72]. Other reports showed that in vivo administration of a dual agonist FXR/TGR5 improved the inflammatory state through immunomodulation of monocytes and macrophages in obese *db*/*db* mice [73]. Furthermore, in cholestatic conditions, TGR5 KO mice were more susceptible to liver injury after a bile duct ligation [74,75]. The reduced TGR5 activation capacity occurring consequently to the UDCA feeding could therefore explain the lack of anti-inflammatory effects of UDCA in C26 mice.

In contrast to previous work where UDCA treatment showed a trend toward attenuation of tissue loss in the rat Yoshida hepatoma model [76], we observed a significant drop in the tibialis weight in C26-UDCA mice. Interestingly, Sasaki and colleagues have recently shown using gain- and loss-of-function models that TGR5 can foster muscle differentiation and hypertrophy while reducing the ubiquitin-proteasome pathway [44]. In contrast, Abrigo and colleagues showed that TGR5 is mandatory for the in vitro pro-atrophy effect of DCA and CA [77]. An exploration of these pathways revealed that, in C26 mice, UDCA reinforces the induction of the genes involved in the ubiquitin-proteasome pathway without affecting key markers of muscle differentiation and hypertrophy. Altogether, these results lead us to exclude a contribution of the TGR5 pathway to the altered myogenic program in C26 mice and to speculate that the selective reduction in TGR5 activation capacity by UDCA in C26 mice may contribute to the exacerbation of the muscle atrophy found in C26 mice only, and vice versa, that TGR5 agonists may hold anti-atrophy therapeutic potential in cancer cachexia.

## 5. Conclusions

With this study, we brought to light important pieces of evidence sustaining the emerging link between bile acids and metabolic disorders associated with cancer. First, we established that bile flow is reduced in this mouse model of cancer cachexia. Secondly, we demonstrated unequivocally that alterations in the bile acid pathways and profile are directly linked to cachexia and cannot be ascribed only to the tumoral presence. Third, we revealed that UDCA, a choleretic compound, does not improve hepatic inflammation and worsens muscle atrophy in cachectic mice. Whether TGR5 could represent an innovative therapeutic target in cancer cachexia has not been formally demonstrated so far and will constitute the focus of our future experimental work.

## Data Availability

No new data were created or analyzed in this study. Data sharing is not applicable to this article.

## Supplementary Materials

The following are available online at https://www.mdpi.com/article/10.3390/cancers13246389/s1. Figure S1: TGR5 activation capacity incubated with individual bile acids and mouse portal plasma with or without TGR5 plasmid, Figure S2: Hepatic TGR5 agonist levels correlate with TGR5 activation capacity in mouse portal plasma, Figure S3: Tumor, liver, brown adipose tissue weights and hepatic bile acid profile in cachectic mice treated with ursodeoxycholic acid, Table S1: The primer sequences for the targeted mouse genes.
