# Supplementary material for: Bile Acid Dysregulation Is Intrinsically Related to Cachexia in Tumor-Bearing Mice
Source: Cancers (Basel). 2021 Dec 20;13(24):6389. doi: 10.3390/cancers13246389 (PMC8699129; doi:10.3390/cancers13246389)
Supplement: Supplementary file 1 [file cancers-13-06389-s001.zip › cancers-1405210-supplementary.pdf]

cancers

MDPI

Supplementary Materials: Bile Acid Dysregulation Is Intrinsically Related to Cachexia in Tumor-Bearing Mice

Morgane M. Thibaut, Justine Gillard, Adeline Dolly, Martin Roumain, Isabelle A. Leclercq, Nathalie M. Delzenne, Giulio G. Muccioli and Laure B. Bindels

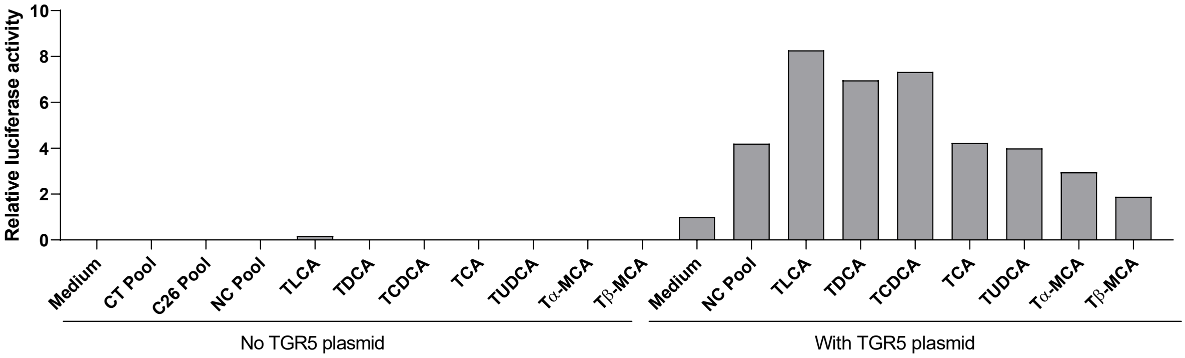

Figure S1. TGR5 activation capacity incubated with individual bile acids and mouse portal plasma with or without TGR5 plasmid. TGR5 activation capacity using cell reporter assay incubated with FBS-free medium (Medium) or FBS-free medium containing 10 µM of tauro-lithocholic acid (TLCA), tauro-cholic acid (TCA), tauro-chenodeoxycholic acid (TCDCA), tauro-α-muricholic acid (Tα-MCA), tauro-β-muricholic acid (Tβ-MCA), tauro-deoxycholic acid (TDCA) and tauro-ursodeoxycholic acid (TUDCA) or pool of 10% portal plasma sham-injected mice (CT), mice injected with cachexia-inducing C26 colon carcinoma cells (C26) and mice injected with non-cachexia-inducing C26 colon carcinoma cells (NC) in cells transfected with pGL4.29 and pGL4.73, with or without pCMV-SPORT6 human TGR5 for 3 hours. Each condition was performed in three technical replicates, excepted for NC Pool (in two replicates).

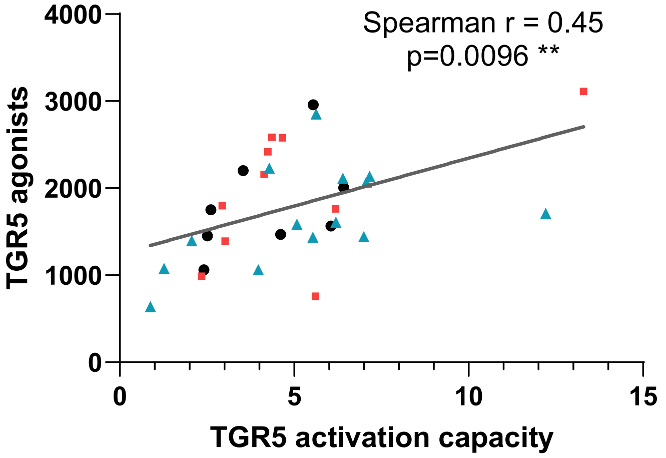

Figure S2. Hepatic TGR5 agonist levels correlate with TGR5 activation capacity in mouse portal plasma. Correlation between hepatic TGR5 agonist levels and TGR5 activation capacity in portal plasma of sham-injected mice (CT; black spots; $n = 8$), mice injected with cachexia-inducing C26 colon carcinoma cells (C26 mice; red squares; $n = 10$) and mice injected with non-cachexia-inducing C26 colon carcinoma cells (NC mice; blue triangles; $n = 14$). ** $p < 0.01$.

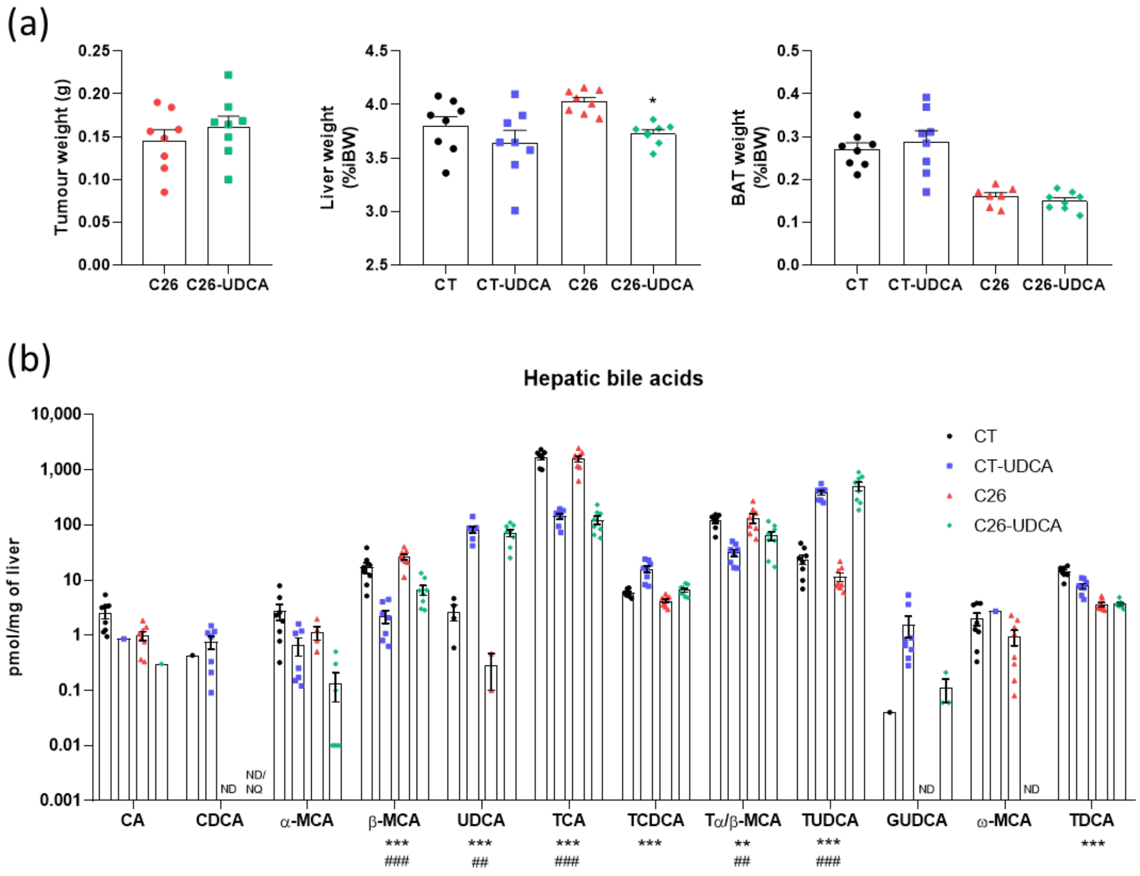

Figure S3. Tumor, liver, brown adipose tissue weights and hepatic bile acid profile in cachectic mice treated with ursodeoxycholic acid. (**a**) Tumor, liver and brown adipose tissue weights of sham-injected mice (CT), sham-injected mice treated with UDCA (CT-UDCA), mice injected with cachexia-inducing C26 colon carcinoma cells (C26) and mice injected with cachexia-inducing C26 colon carcinoma cells and treated with UDCA (C26-UDCA). (**b**) Hepatic bile acids in CT, CT-UDCA, C26 and C26-UDCA mice. n = 7-8 mice per group; data are presented as mean ± SEM. ** $p < 0.01$, *** $p < 0.001$ CT vs CT-UDCA and # $p < 0.05$ ## $p < 0.01$, ### $p < 0.001$ C26 vs C26-UDCA. ND, Not detected; NQ, Not quantified.

Table S1. The primer sequences for the targeted mouse genes.

Target name	Alias/full name	Forward	Reverse
Rpl6	Ribosomal Protein L6	CTTGTGACTGGGCCTCTTGT	GCGTCAGTCAGGTGTTTGG
Oaz1	ornithine decarboxylase antizyme 1	TCATCTGCTTCCCCAAGAAC	TGGCACCTAGTCCTCCTCAC
Ntcp	Na(+)/taurocholate transport protein	GGACAAGGTGCCCTACAAAG	ACAGCCACAGAGAGGGAGAA
Oatp1b2	organic anion transporter family member 1B2	ATCCCGTGACTAATCCAACA	ACCAAACTGCTGCTCTATAAACT
Bsep	bile salt export pump	AGATACAACCGAAGGGGACA	TCAACTTCTTCCACAAGCACA
Mrp2	Multidrug resistance-associated protein 2	GGATGGTGACTGTGGGCTGAT	GGCTGTTCTCCCTTCTCATGG
Ostβ	Organic solute transporter subunit beta	GTATTTTCGTGCAGAAGATGCG	TTTCTGTTTGCCAGGATGCTC
Cyp7a1	cytochrome P450 family 7 subfamily A member 1	GGGATTGCTGTGGTAGTGAGC	GGTATGGAATCAACCCGTTGTC
Cyp8b1	cytochrome P450 family 8 subfamily B member 1	GATCCGTCGCGGAGATAAGG	CGGGTTGAGGAACCGATCAT
Cyp27a1	cytochrome P450 family 27 subfamily A member 1	TCTGGCTACCTGCACTTCCT	GTGTGTTGGATGTCGTGTCC
Dio2	iodothyronine deiodinase 2	AATTATGCCTCGGAGAAGACCG	GGCAGTTGCCTAGTGAAAGGT
Ucp1	uncoupling protein 1	GCTACACGGGGACCTACAATG	CGTCATCTGCCAGTATTTTGTT
Acox1	acyl-CoA oxidase 1	CTATGGGATCAGCCAGAAAGG	AGTCAAAGGCATCCACCAAAG
Cidea	cell death inducing DFFA like effector a	TGGAAAAGGGACAGAAATGG	TCTCGTACATCGTGGCTTTG
Gk	glycerol kinase	TTTGTTTCCAAACCCGAGAG	AGCGCCTAGTGCAGTTGTTT
Il1β	interleukin-1β	TCGCTCAGGGTCACAAGAAA	CATCAGAGGCAAGGAGGAAAAC
Ccl2	C-C motif chemokine ligand 2	GCAGTTAACGCCCCACTCA	TCCAGCCTACTCATTGGGATCA
Cxcl1	C-X-C motif chemokine ligand 1	AAACCGAAGTCATAGCCACAC	AAGCCAGCGTTCACCAGA
Cxcl2	C-X-C motif chemokine ligand 2	CGCCCAGACAGAAGTCATAG	CTCCTCCTTTCCAGGTCAGTT
Icam1	intercellular adhesion molecule 1	GAGAGTGGACCCAACTGGAA	CACACTCTCCGGAAACGAA
Mmp8	matrix metallopeptidase 8	CCTTCCTACCCAACGGTCTT	GCCCAGTACTGTCTGCCTTT
Vcam1	vascular cell adhesion molecule 1	GAACCCAAACAGAGGCAGAG	CCATCACTTGAGCAGGTCAG
Nlrp3	NLR family pyrin domain containing 3	GCCCAAGGAGGAAGAAGAAG	AGAAGAGACCACGGCAGAAG
Trim63	Tripartite Motif Containing 63	ACGAGAAGAAGAGCGAGC	CTTGGCACTTGAGAGGAA
Fbxo32	F-Box Protein 32	ATGCACACTGGTGCAGAGAG	TGTAAGCACACAGGCAGGTC
Musa1	muscle ubiquitin ligase of the SCF complex in atrophy 1	TTCCTTGACGGCTTCAGTTT	TGAATCGCCATACCTTCTCTTT
Map1lc3a	Microtubule Associated Protein 1 Light Chain 3 Alpha	CACTGCTCTGTCTTGTGTAGGTTG	TCGTTGTGCCTTTATTAGTGCATC
Ctsl	Cathepsin L	GTGGACTGTTCTCACGCTCAAG	TCCGTCCTTCGCTTCATAGG
MyoG	myogenin	CAGTGAATGCAACTCCCACA	GGCAACAGACATATCCTCCA
MyoD	myogenic differentiation 1	TCCCTAAGCGACACAGAACA	CGAAAGGACAGTTGGGAAGA
Ucp2	uncoupling protein 2	GGTCGGAGATACCAGAGCAC	TGAGGTTGGCTTTCAGGAGA
Pax7	paired box 7	AAAGCCAAACACAGCATCG	GTCGGGTTCTGATTCCACAT
Igf1	insulin like growth factor 1	GCTCTTCAGTTCGTGTGTGG	CACAATGCCTGTCTGAGGTG
Ppargc1a	PPARG coactivator 1 alpha	AGCCGTGACCACTGACAACGAG	GCTGCATGGTTCTGAGTGCTAAG
